# White Piedra in a Mother and Daughter

**DOI:** 10.4103/0974-7753.58559

**Published:** 2009

**Authors:** Anupama S Roshan, C Janaki, B Parveen

**Affiliations:** Department of Dermatology, Madras Medical College, Chennai, India

**Keywords:** Topical terbinafine 1%, *Trichosporon* spp, White Piedra

## Abstract

White Piedra is a superficial fungal infection of the hair caused by *Trichosporon asahii*. It is also known as trichomycosis nodosa or trichomycosis nodularis. We report two cases of White Piedra in a mother and her daughter for the rarity of such occurrence.

## INTRODUCTION

White Piedra is an unusual asymptomatic superficial fungal infection of the hair, characterized by the presence of numerous, discrete, soft, asymptomatic nodules loosely attached to the infected hair shafts. It may occur on the scalp, eyebrows, eyelashes, beard, axilla or in the groin. Compared with Black Piedra, which almost always occurs on the scalp hair, White Piedra less commonly affects the scalp hair and is more common on other hairy sites of the body. It is caused by a yeast-like fungus, *Trichosporon beigelii*, now known as *T. asahii*,[1] first described by Beigel in 1865. This is more common in temperate climates and has been reported in Europe and North and South America. Although it has been reported from many parts of Asia, it is less common in the tropics. Successful treatment of White Piedra has been achieved with clipping of affected hair or tonsuring and use of topical antifungal agents.

## CASE REPORT

**Figure 1:**
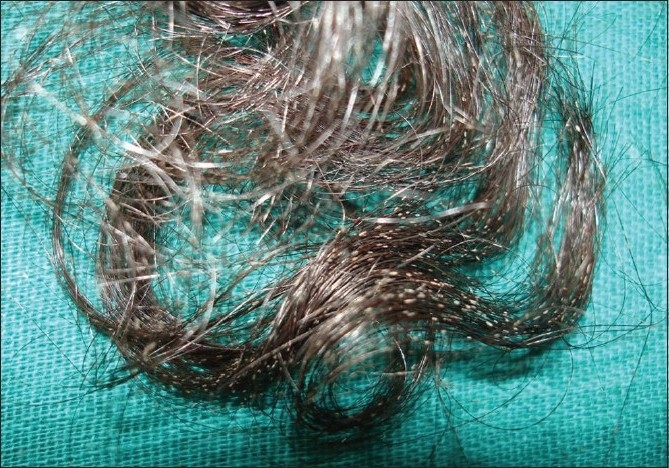
Whitish to cream-colored easily detachable nodules of size 1-1.5 mm present over the shaft of almost all the scalp hair

A 45-year-old lady presented with asymptomatic whitish nodules in the scalp hair for the past 3 years along with breakage of the hair shafts [Figure 1]. Her 20-year-old daughter also had similar complaints for the past 6 months. Both of them had the habit of tying their wet hair up in a knot after a hair wash and shared a common comb. There was no pediculosis in either of them.

Clinical examination revealed whitish to cream-colored, easily detachable nodules of size 1-1.5 mm present over the shaft of almost all the scalp hairs. The nodules were seen as encircling the hair shaft completely. Scalp skin and other hairy areas were normal.

**Figure 2:**
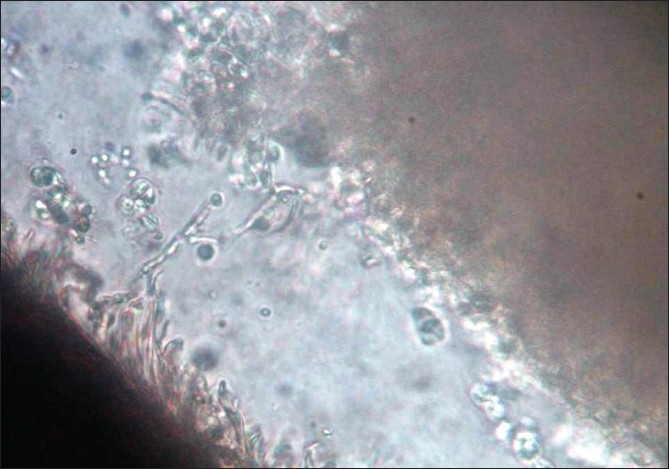
KOH 10% wet mount of the affected hairs showed septate hyaline hyphae arranged perpendicular to the hair shaft

**Figure 3:**
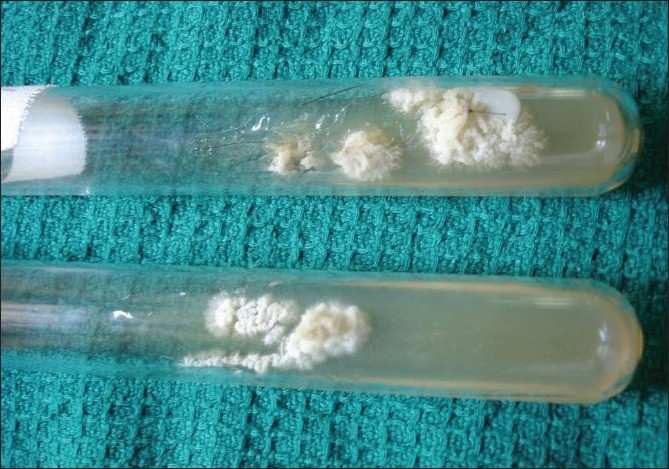
Culture (in agar without cycloheximide) grew yeast-like creamy white colonies after 48 h of incubation

**Figure 4:**
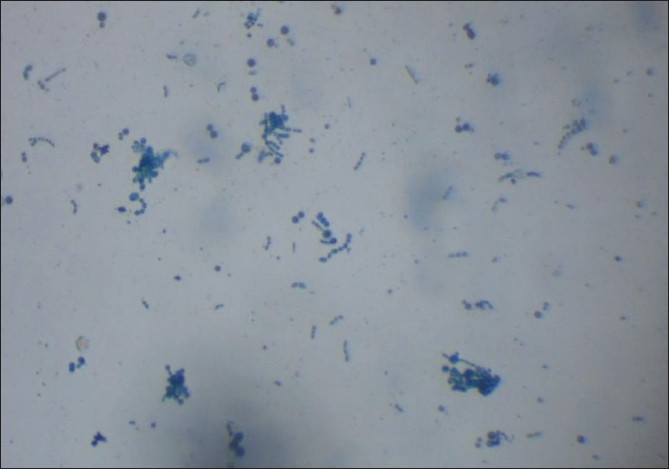
Lactophenol cotton blue mount showing septate hyphae, arthroconidia and budding blastoconidia

Hematological and biochemical investigations were within normal limits. Test for human immunodeficiency virus infection was negative. Potassium hydroxide (KOH 10%) wet mount of the affected hairs showed septate hyaline hyphae arranged perpendicular to the hair shaft. Arthrospores and blastospores were seen [Figure 2]. Culture in Sabourauds dextrose agar (without cycloheximide) grew yeast-like creamy-white colonies after 48 h of incubation [Figure 3]. Later, the colonies became wrinkled, convoluted and had a heaped-up center. The colonies were creamy white on the reverse. Lactophenol Cotton Blue mount showed septate hyphae, arthroconidia and budding blastoconidia [Figure 4]. Thus, the cultural characteristics confirmed Trichosporon spp. although speciation was not possible. Both the patients were advised to keep the hair dry and were treated with topical application of 1 in 2,000 mercuric perchloride for 3 months along with trimming of the hair regularly. They were also advised topical terbinafine (1%) twice daily for the next 3 months, with total resolution of the nodules. Both the mother and the daughter were followed for the next 6 months, during which time there was no relapse.

## DISCUSSION

Human White Piedra may affect hairs of the scalp, axilla or crural areas. White Piedra of the scalp occurs with a low incidence in tropical and subtropical countries.[2,3] People of all age groups are affected, with a higher incidence in young women. It is characterized by soft white nodules similar to nits but can be easily pulled off, unlike nits. The nodules may be white, pale green or yellow and are composed of compact fungal elements. The hairs are not invaded but they may break if the fungi have been there for long period, as has happened in our cases. Occasionally, the adjoining skin can get infected, especially in areas like the groin, causing intertrigo, which is considered as a form of cutaneous trichosporosis.[4]

White Piedra is caused by several Trichosporon species, like *T. asahii, T. cutaneum, T. inkin, T. ovoides* and *T. mucoides*. Members of this species have multilamellar cell walls in common and contain more or less developed dolipore septa with or without vesicular or tubular parenthesomes.[5] Budding cells are abundant in primary cultures but hyphae predominate after repeated transfer.

Our patient gave a typical history of tying up wet hair and also sharing of comb with her daughter, enabling disease transmission. The disorder can be controlled by shaving and by local application of 5% ammoniated mercury ointment, topical 2% miconazole, 2% ketoconazole or 1% terbinafine four times a day for a period of 2 weeks or till remissions occur.[1] Oral itraconazole therapy has also been suggested.[6] Although it relapses frequently, removal of the affected hair is usually curative, with few recurrences.
